# Associations among Inflammatory Biomarkers in the Circulating, Plasmatic, Salivary and Intraluminal Anatomical Compartments in Apparently Healthy Preschool Children from the Western Highlands of Guatemala

**DOI:** 10.1371/journal.pone.0129158

**Published:** 2015-06-15

**Authors:** María José Soto-Méndez, María Eugenia Romero-Abal, Concepción María Aguilera, María Cruz Rico, Noel W. Solomons, Klaus Schümann, Angel Gil

**Affiliations:** 1 Center for the Studies of Sensory Impairment, Aging, and Metabolism–CeSSIAM- Guatemala City, Guatemala; 2 Department of Biochemistry and Molecular Biology II, Institute of Nutrition and Food Technology, Center of Biomedical Research, University of Granada, Granada, Spain; 3 Molecular Nutrition Unit, ZIEL, Research Center for Nutrition and Food Science, Technische Universität München, Freising, Germany; Penn State College of Medicine, UNITED STATES

## Abstract

**Background:**

Undernutrition and inflammation are related in many ways; for instance, non-hygienic environments are associated with both poor growth and immunostimulation in children.

**Objective:**

To describe any existing interaction among different inflammation biomarkers measured in the distinct anatomical compartments of whole blood, feces, plasma and saliva.

**Methods:**

In this descriptive, cross-sectional study, samples of whole blood, feces, plasma and saliva were collected on the 8^th^ and last week of observation among 87 attendees (42 girls and 45 boys) of 3 daycare centers offering a common 40-day rotating menu in Guatemala’s Western Highlands. Analyses included white blood cell count (WBC), fecal calprotectin, and plasmatic and salivary cytokines including IL-1B, IL-6, IL-8, IL-10 and TNF-α. Associations were assessed using Spearman rank-order and goodness-of-fit correlations, as indicated, followed by backwards-elimination multiple regression analyses to determine predictor variables for IL-10 in both anatomical compartments.

**Results:**

Of a total of 66 cross-tabulations in the Spearman hemi-matrix, 22 (33%) were significantly associated. All 10 paired associations among the salivary cytokines had a significant r value, whereas 7 of 10 possible associations among plasma cytokines were significant. Associations across anatomical compartments, however, were rarely significant. IL-10 in both biological fluids were higher than corresponding reference values. When a multiple regression model was run in order to determine independent predictors for IL-10 in each anatomical compartment separately, IL-6, IL-8 and TNF-α emerged as predictors in plasma (r^2^ = 0.514) and IL-1B, IL-8 and TNF-α remained as independent predictors in saliva (r^2^ = 0.762). Significant cross-interactions were seen with WBC, but not with fecal calprotectin.

**Conclusion:**

Interactions ranged from robust within the same anatomical compartment to limited to nil across distinct anatomical compartments. The prominence of the anti-inflammatory cytokine, IL-10, in both plasma and saliva is consistent with its counter-regulatory role facing a broad front of elevated pro-inflammatory cytokines in the same compartment.

## Introduction

The immune system, in its complexity, is constituted of specialized cells with specific secretory or functional roles. There are the rapid “innate” and the slower “adaptive” immune responses, highly integrated by hormonal signaling or cell-to-cell cross-talk [1]. These mobilize cellular elements, including, phagocytic, inflammatory and natural killer cells, among others, along with molecular components, such as hepatic acute-phase proteins and cytokines originating from a wide variety of tissues. The cytokines produced by white blood cells constitute a series of “interleukins.” Differentiation of thymic-derived helper cells produces subclasses supporting up-regulating, pro-inflammatory cytokines (Th1) and counter-reacting, down-regulating anti-inflammatory cytokines (Th2). [1]. The Th1-directed acute-phase response directs a catabolic intermediary metabolism, tending toward poor tissue growth and wasting of nutrients [2].

Guatemala has the highest prevalence of under-five stunting in Latin America [3]. Poor linear growth begins in utero [4,5], and continues during the first 2 y of life [6]. Stunting generates a series of adverse consequences not only in infancy and childhood, but also over the longer term; as summarized by Dewey and Begum [7]: “childhood stunting (was) linked with short adult stature, reduced lean body mass, less schooling, diminished intellectual functioning, reduced earnings and lower birth weight of infants born to women who themselves had been stunted as children.” In the context of the complexity of immune reactivity, linear growth impairment is associated with immunological alterations such as impaired gut-barrier function, reduced delayed-type hypersensitivity responses, atrophy of lymphatic tissue whereas the cytokine patterns seem to be skewed towards a Th2-response [8], as part of a well-described cytokine-induced or infection-induced malnutrition [9].

It has long been established in livestock and poultry [10,11] that unclean and contaminated environments retard body growth and weight-gain. Roura et al. [12] identified a cytokine-mediated immunological stress as the mechanism for this growth failure. In 1993, Solomons, et al. [13] proposed an explanation for poor linear growth based on the theory of environmental contamination and poor utilization of nutrients. It has been proposed that decreasing inflammatory episodes will improve long-term outcomes on linear growth [14]. Interventions to prevent environmental enteropathy during infancy such as WASH (Water, Sanitation and Hygiene) in Kenya and Bangladesh or SHINE (Sanitation Hygiene Infant Nutrition Efficacy) in Zimbabwe suggest that low-grade, chronic inflammation may impair infant growth and that reducing fecal-oral transmission of pathogenic microbes during infancy will reduce prevalence of stunting in developing countries [15,16]. A plausible mechanism for the direct interference with linear growth by infection comes from the work with an infected-mouse model [17], in which endogenous stress compounds (IL-1B, cortisol) interrupt the hormonal cascade to the epiphyseal growth plate that signals elongation of bone. Hence, linear growth retardation is not only due to undernutrition or lack of nutrients; additionally, continuous inflammation of the body promotes the malabsorption and wasting of nutrients and dysregulation of skeletal growth.

In the course of a research project entitled “Study on the normative state and inter- and intra-individual variation in growth, hematology, hydration, and markers of oxidation, infection and inflammation in pre-school children with a similar dietary intake”, we collected data on white blood cells, a biomarker of intraluminal intestinal inflammation, and a parallel series of selected cytokines in plasma and saliva among preschool children in a governmental system of daycare centers. We attempted to find any possible interaction among the inflammatory biomarkers from different anatomical compartments as an example of immunological cross-talk and reafferent integration of the system. We present here the findings from this exploration in young children within the context of variable environmental and genetic circumstances, within the potentially stabilizing and harmonizing influence of a common institutional dietary offering.

## Materials and Methods

### Study Design

The following descriptive, cross-sectional, field study on the variation and associations among variables related to inflammation is part of the larger undertaking entitled: “Study on the normative state and inter- and intra-individual variation in growth, hematology, hydration, and markers of oxidation, infection and inflammation in pre-school children with a similar dietary intake.”

### Population and Setting

The study was conducted in Guatemala in the Western Highlands Province of Quetzaltenango, known for its rural-based agrarian environment. With its capital city located 220 km from Guatemala City at 2357 m above sea level, Quetzaltenango Province has a majority indigenous population (60.6%) and an annual mean daily low temperature of 14.7°C, but varying from -12 to 25°C.

Study subjects were children attending daycare centers (*Hogares Comunitarios)* within the Secretariat of Beneficial Programs of the First Lady (*Secretaría de Obras Sociales de la Esposa del Presidente-* SOSEP) in three different settings: one semi-urban, one marginal-urban, and one rural. Each site had differences in its proportional ethnic make-up and the corresponding cultural customs and traditions. The rural site had an almost exclusively *Mam*-Mayan indigenous enrolment. SOSEP daycare centers offer a common, 40-day rotating menu. It is standardized in its recipes and provides four meals per attendance day.

### Recruitment and Enrolment of Subjects

Children from three daycare centers were eligible to enroll and be included in the analyses if they were: 1. attending one of the centers; 2. between 2 and 7 years-old; and 3. (post-hoc) had at least an 80% daily attendance record in the center during the time of the study. Moreover, they had to be apparently healthy and without restrictions related to the acceptance of the diet offered in the SOSEP menu. Children whose parents or caregivers did not sign the consent form, or who did not adhere to the full fecal collection schedule were selectively excluded from the analyses.

### Ethical Considerations

The SOSEP’s director for the Quetzaltenango area authorized the study to be performed within the system. The Human Subjects Committee of the Centre for the Studies of Sensory Impairment, Aging, and Metabolism (CeSSIAM) granted ethical approval for the study protocol. A parent or guardian signed the written consent form after the purposes, benefits, inconveniences and risks of the procedures had been explained. Children gave a final assent at the moment of collection. As a collective benefit, any missing dietary items were subsidized by the study funds in order to provide all food items on the menu at all times, whenever the situation required. This study was registered at clinicaltrials.gov as NCT02203890.

### Anthropometric Measurements

We performed anthropometric determinations during the last 7^th^ week of each daycare center’s 8-week period. Height was measured using a wooden wall stadiometer, and expressed in cm to the nearest 0.5 cm, with children standing without shoes and with their gaze in Frankfort plane. Weight was determined using a calibrated Tanita Model BC522 digital scale (Tanita, Tokyo, Japan); still with shoes removed. It was expressed in kg to the nearest 0.1 kg. An adjustment was made for clothing, subtracting a standard weight-for-clothing factor for girls’ and boys’ daytime clothes, respectively.

### Collection, Handling and Storage of Biological Samples

In order to confirm the delivery of the 40-day rotating menu, we spent 8 weeks on each day-care center as an observation period from June to November, 2012, with sample collections beginning in July and extending to the end of the field activities. Whole blood assays (white blood cell count) were performed on the day of blood collection. Fecal samples were frozen-stored for from 3 to 16 weeks prior to analytic processing. Plasma and saliva samples, destined for cytokine assays, were frozen-stored for up to one year prior to the analyses in Spain.

#### Blood (plasma and whole blood)

Blood samples were collected at each of the three centers during the last week of the 8-week study. A phlebotomist extracted the blood using BD Vacutainer 4 mL tubes, anticoagulated with EDTA (No.367861) in conjunction with Safety-Lok deposable needles (No.367281) (Becton-Dickinson, NJ, USA). Five hundred microliters of the sample were separated in a tube and taken to *La Democracia* Hospital’s clinical chemistry laboratory in order to obtain hemograms. The rest of each sample was centrifuged to separate red blood cells from plasma; the supernatant plasma was stored in Nalgene Cyrogenic Vials (No.5000-0012) (U.S. Plastics Corporation, Lima, OH, USA) in a -80°C freezer in the capital city prior to shipment to Granada, Spain, in order to measure plasma cytokines like interleukin-1-beta (IL-1B), interleukin-6 (IL-6), interleukin-8 (IL-8), interleukin-10 (IL-10) and tumor necrosis factor-alpha (TNF-α).

#### Fecal samples

In the 7^th^ week of the 8-week period, we supplied a container to the parents or caregivers to collect a fecal sample on the next morning, before bringing the child to the daycare center. Whenever the container was returned empty, we collected the sample if it was produced during the day; we repeated the process until we had all participants’ samples. When samples were complete, they were taken to the local laboratory where we started the pre-preparation of the specimens to measure calprotectin; these were stored in a -20°C freezer, ready for biomarker assaying.

#### Saliva

On the day of blood collection, we also collected a saliva sample. Children were asked not to eat or drink anything during the two hours before saliva collection. Saliva was immediately stored in dry ice until storage in a -80°C freezer in the capital city and later shipped (again in dry ice) to Granada, Spain, in order to perform a parallel set of cytokine assays in both plasma and saliva.

### Laboratory Assays and Analyses

#### White blood cell count

Analyses were performed in Quetzaltenango, Guatemala, at the *La Democracia* Hospital’s clinical chemistry laboratory, using the Beckman Coulter AcT Diff Hematology Analyzer (Krefeld, Germany). White blood cell counts are expressed as quantity per mm^3^ volume.

#### Fecal Calprotectin

Assays were performed in Quetzaltenango, Guatemala. ELISA assays were executed using the CALPRO Calprotectin ELISA Test from CALPRO AS (Lysaker, Norway). Catalog No. CAL0100. Concentrations were expressed in mg/kg of fecal sample. The detection limits were 25–2500 mg/kg, and a typical inter-assay CV was 3.7%.

#### Plasma and Saliva Cytokines

Samples were analyzed in Granada, Spain, using the MILLIPLEX MAP Human High Sensitivity Cytokine panel from Luminex Corporation (Missouri, USA) Catalog # HSCYTMAG-60SK for the five aforementioned classes of cytokines. The results were expressed in pg/mL. The cytokines of interest for both plasma and saliva were: IL-β, IL-6, IL-8, IL-10 and TNF-α. The minimum detection limit was 0.06, 0.20, 0.05, 0.48 and 0.07 respectively. The inter-assay CVs for plasma were 14.73%, 7.74%, 8.65%, 12.17% and 8.40%, respectively. For salivary cytokines CVs were 10.99%, 13.88%, 13.31%, 7.74%, 10.99% and 13.34%, respectively.

### Data Handling and Statistical Analyses

The software SPSS Version 20 was used to create a database and run analyses. Descriptive statistics were expressed as the distribution in terms of median, 95% CI, and minimum and maximum. Associations of values collected at different points in time were tested by Spearman rank-order coefficient, as appropriate. We also ran the goodness-of-fit model to obtain correlation coefficients. In order to refine the predictive determination in the associations among the inflammation biomarkers, backwards-elimination multiple regression models were run to determine the parsimonious r^2^ value. A probability level of ≤0.05 was accepted as statistically significant. STROBE statement for this article is included as supporting information file (**S1 Checklist**).

## Results

### Characteristics of the Participants

**Fig 1:**
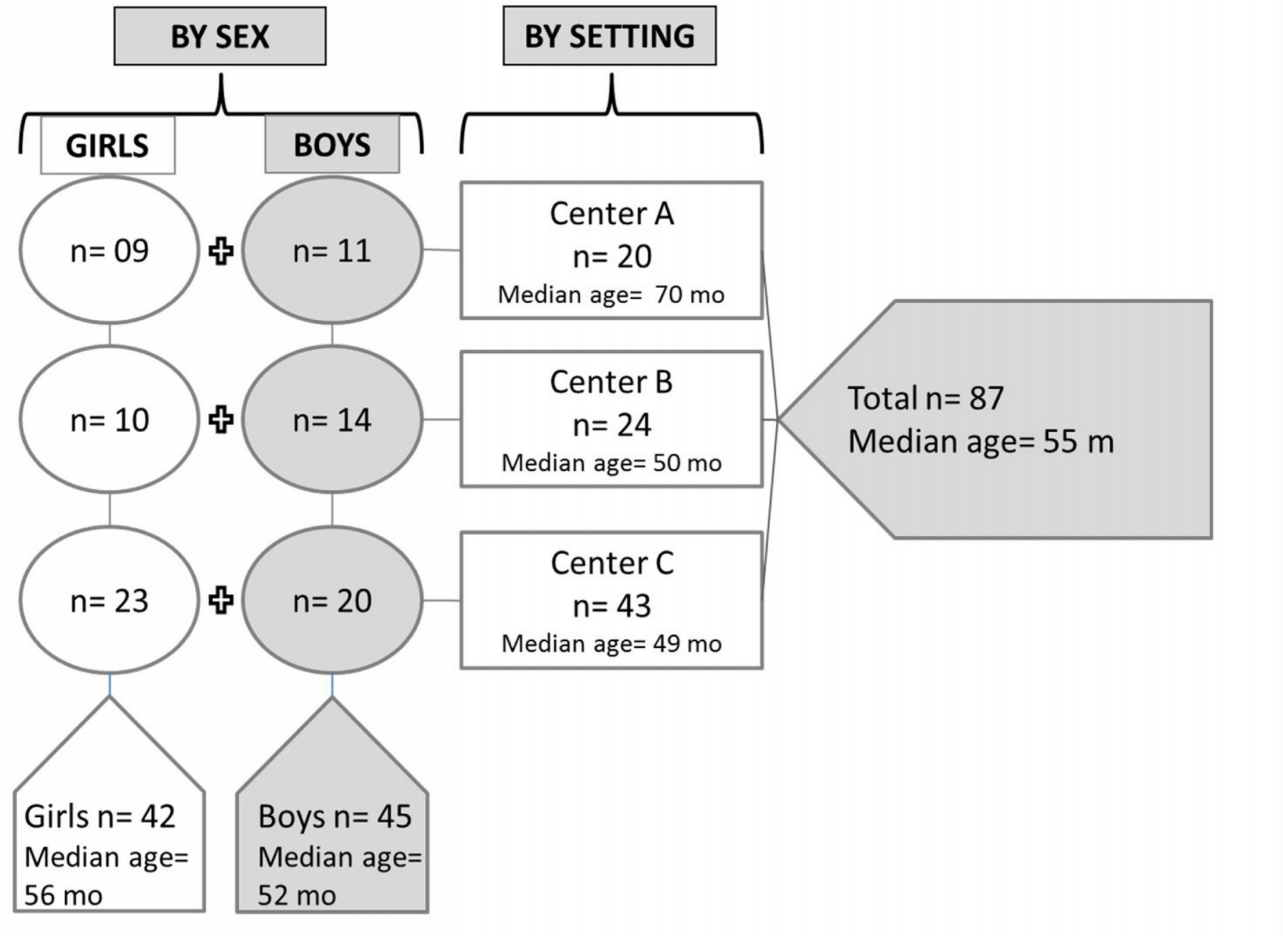
Characteristics of the Subjects. Legend: Characteristics expressed for the overall sample and disaggregated by setting and sex. This includes the respective numbers as well as the median age for each grouping. The data for girls and for the overall sample are included in the clear areas and those for boys and overall sample in the shaded areas.

**Fig 2:**
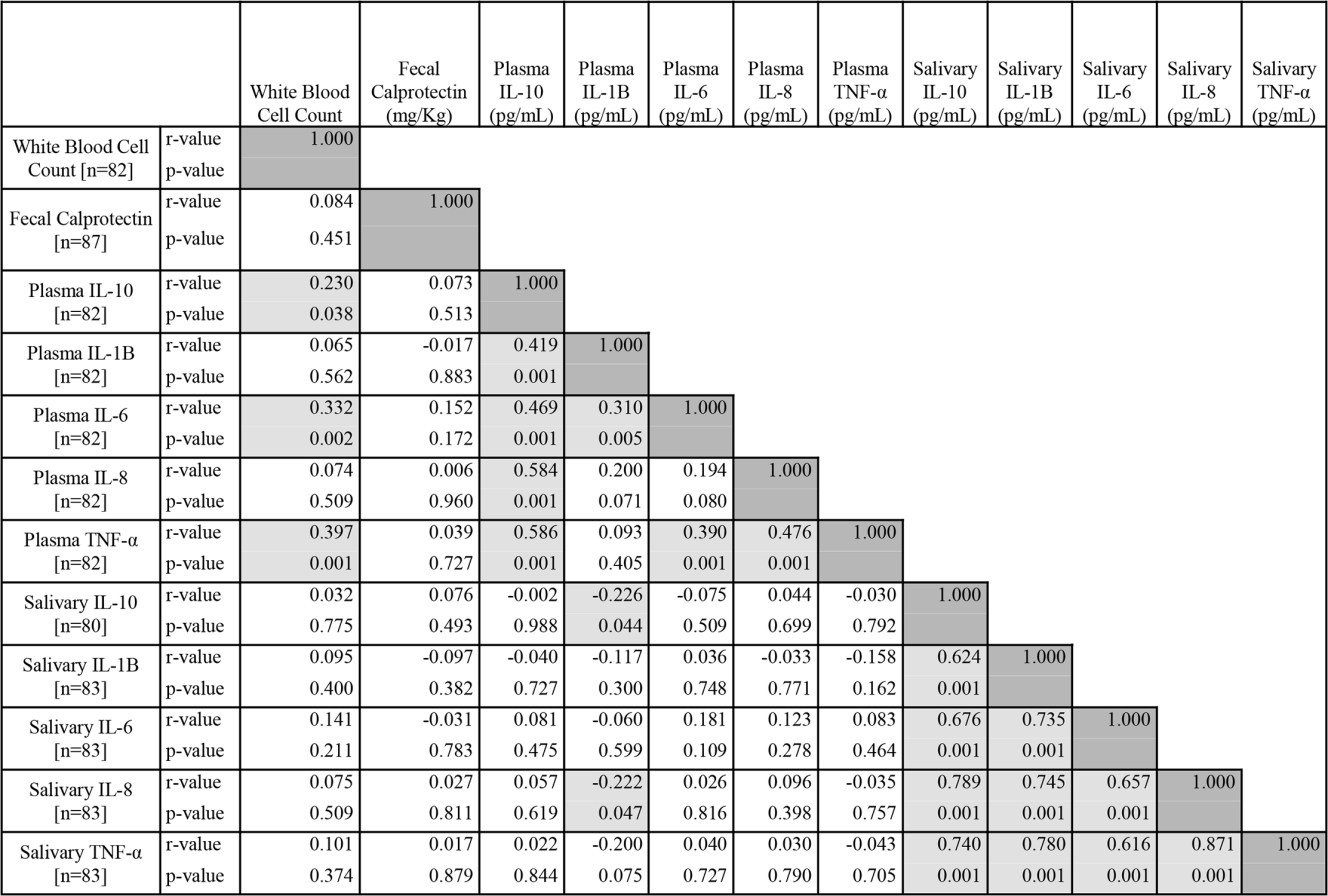
Spearman Correlation Coefficient Hemi-Matrix for Inter-Relationships of Biomarkers. Legend: The Spearman rank-order correlation coefficients Hemi-Matrix for mutual, cross inter-relationships of the 12 measured biomarkers is illustrated within the 78 cells of pertinent reference. The dark-shaded cells represent the 12 auto-correlations. The remaining 66 cells illustrate the probability level for the corresponding Spearman r value. The 22 medium-shaded cells have statistically-significant associations, whereas the 44 clear cells have non-significant associations. Footnotes: The units of expression for concentrations are provided in the superior captions on the x-axis. The numbers of individuals with analyzed values are provided in the left-hand column y-axis.

Overall, 87 children had at least one inflammation datum (**Fig 1**). These included 42 girls and 45 boys. They had a median age of 55 mo, with a mean of 54 ± 16 mo and ranged from 23 to 81 mo. **Fig 1** also disaggregates the sample by site and sex. Table 1 presents the data on growth and nutritional status derived from the anthropometric measurements. Illustrated are the Z-scores for HAZ, WAZ and WHZ, and the respective prevalence of stunting, underweight and wasting for the entire sample and the distinct geographic sites. It was possible to make binary pairing of inflammation data for from 80 to 87 children, depending on the combinations (**Fig 2**).

**Table 1:** Median Height-for-Age, Weight-for-Age and Weight-for-Height of the Subjects, overall and disaggregated by daycare center.

		Height-for-Age (stunting)	Weight-for-Age (underweight)	Weight-for-Height (wasting)
Setting	N	Z-score (% subnormal)		
Total Sample	87	-2.39 (66%)	-1.37 (23%)	-1.00 (2%)
Semi-Urban (A)	20	-1.61^a^ (35%)	-0.96^a^ (0%)	-0.10 (0%)
Marginal-Urban (B)	24	-2.42^ab^ (63%)	-1.46^b^ (17%)	-0.16 (4%)
Rural-(C)	43	-2.73^b^ (81%)	-1.47^b^ (37%)	-0.07 (2%)
p-value		0.004*	0.025*	0.434*

*Comparison among settings (last three rows) using the ANOVA test.

Values not sharing the same superscript were statistically different by the Bonferroni post-hoc test

### Descriptive statistics of the inflammatory biomarkers

Illustrated in **Table 2** are median, 95% CI, and limits for each of the 12 inflammatory biomarkers data set, grouped by origin: whole blood; feces; plasma; and saliva. In descending order, the concentrations for plasma values are: IL-10; TNF-α; IL-8; IL-6; and IL-1B. For saliva, corresponding values are: IL-8; IL-10; IL-1B; IL-6; and TNF-α. Only IL-8 and IL-1B had higher concentrations in saliva as compared to plasma, whereas IL-10; IL-6 and TNF-α had higher concentrations in plasma compared to saliva. In the final column of **Table 2**, we provide reference range values for normative values. A caveat is that the plasma and salivary cytokines, however, are from adult women, 40–50 y older rather than preschool subjects [18].

**Table 2:** Descriptive Statistics of Biomarker Concentrations.

Class	Biomarker	N	Median	95% CI	Min-Max	Reference
Whole blood	White blood cells (thousands/mm^3^)	82	7.6	7.4–8.5	1.8–17.6	3.5–10.5
Fecal	Calprotectin (mg/kg)	87	57.5	69.1–126.9	10.0–950.0	<50^x^
Plasmatic (pg/mL)	IL-1B	82	0.9	0.9–1.2	0.3–5.7	0.37–1.25^y^
	IL-6	82	3.7	3.8–6.1	1.6–44.2	0.52–1.89^y^
	IL-8	82	4.5	4.5–5.6	1.5–13.4	6.5–13.8^y^
	IL-10	82	52.2	52.9–86.5	17.0–608.1	0.60–2.70^y^
	TNF-α	82	7.5	7.5–8.7	4.43–18.0	0.94–2.64^y^
Salivary (pg/mL)	IL-1B	83	1.7	1.2–22.8	0.3–407.1	21.1–73.0^y^
	IL-6	83	1.4	0.8–8.1	0.11–151.2	1.59–10.46 ^y^
	IL-8	83	120	160–256	1.92–734.3	254–578 ^y^
	IL-10	83	11.0	13.8–23.2	1.2–108.8	0.71–3.93^y^
	TNF-α	83	0.8	1.1–2.9	0.1–32.9	4.3–19.0^y^

x = manufacturer’s suggested cut-off for normal calprotectin

y = published normative post-menopausal females’ for cytokines [Browne et al., 2013], expressed as 25^th^ and 75^th^ percentiles

### Inter-Biomarker Associations by Linear (Spearman) Correlations

Of a total of 66 cross-tabulations in the Spearman hemi-matrix **(Fig 2)**, 22 (33%) were significantly associated at the level of p≤0.05. In the face of multiple comparisons with this number of cross-correlations, however, one could expect that at least 3 would reach a probability level of 5% by chance alone. Hence, at least 19 of the associations are likely to be truly significant. The highest degree of linear association, with an r value of 0.871, was salivary IL-8 and salivary TNF-α. However, all 10 paired associations among the salivary cytokines had an r value >0.600 and a probability value of 10^−3^. The lowest r values still reaching the criterion for significance were a pair at 0.222 and 0.226 involving paired correlations of plasma IL-1B with two salivary cytokines; the notable exception was with salivary and plasmatic TNF-α.

### Inter-Biomarker Associations by Goodness-of-Fit Correlations

**Table 3** illustrates the 22 out of 66 Spearman rank-order correlations that reached the ≤0.05 p-value criterion for statistical significance, juxtaposed with the corresponding r value for the goodness-of-fit correlation. Also shown is the curve-form of the goodness-of-fit correlation. The magnitude of the r value improved (increase of >0.020 decimal points of improvement above the Spearman coefficient) in 9 instances (41%), with the greatest increment that of a 50% increase in the value of the association of IL-10 with IL-6 in plasma; r values remained relatively stable (change of -0.020 to +0.020) in 7 cases (32%); and the value declined (decrease of >0.020) in 6 (27%). The cubic curve-form was the predominant one, emerging in 7 regressions, followed by sigmoid and power curve-forms with 6 each. The remaining 3 curve-forms were a growth, a compound, and a logarithmic.

**Table 3:** Comparison of Spearman and Non-Linear Correlation Coefficients in Inter-Biomarker Significant Associations.

X-Axis	Y-Axis	Spearman r-value	Goodness-of-fit r-value	Best model curve form
White Blood Cells	Plasma IL-10	0.230	0.205	Cubic
	Plasma IL-6	0.332	0.323	Sigmoid
	Plasma TNF-α	0.397	0.451	Cubic
Plasma IL-10	Plasma IL-1B	0.419	0.383	Sigmoid
	Plasma IL-6	0.469	0.703	Cubic
	Plasma IL-8	0.584	0.624	Cubic
	Plasma TNF-α	0.586	0.538	Power
Plasma IL-1B	Plasma IL-6	0.310	0.315	Sigmoid
	Salivary IL-10	-0.226	0.257	Power
	Salivary IL-8	-0.222	0.309	Cubic
Plasma IL-6	Plasma TNF-α	0.390	0.378	Sigmoid
Plasma IL-8	Plasma TNF-α	0.476	0.489	Power
Salivary IL-10	Salivary IL-1B	0.624	0.686	Growth
	Salivary IL-6	0.676	0.687	Power
	Salivary IL-8	0.789	0.745	Cubic
	Salivary TNF-α	0.740	0.729	Power
Salivary IL-1B	Salivary IL-6	0.735	0.713	Sigmoid
	Salivary IL-8	0.745	0.707	Logarithmic
	Salivary TNF-α	0.780	0.872	Cubic
Salivary IL-6	Salivary IL-8	0.657	0.707	Sigmoid
	Salivary TNF-α	0.616	0.635	Power
Salivary IL-8	Salivary TNF-α	0.871	0.854	Compound

**Fig 3:**
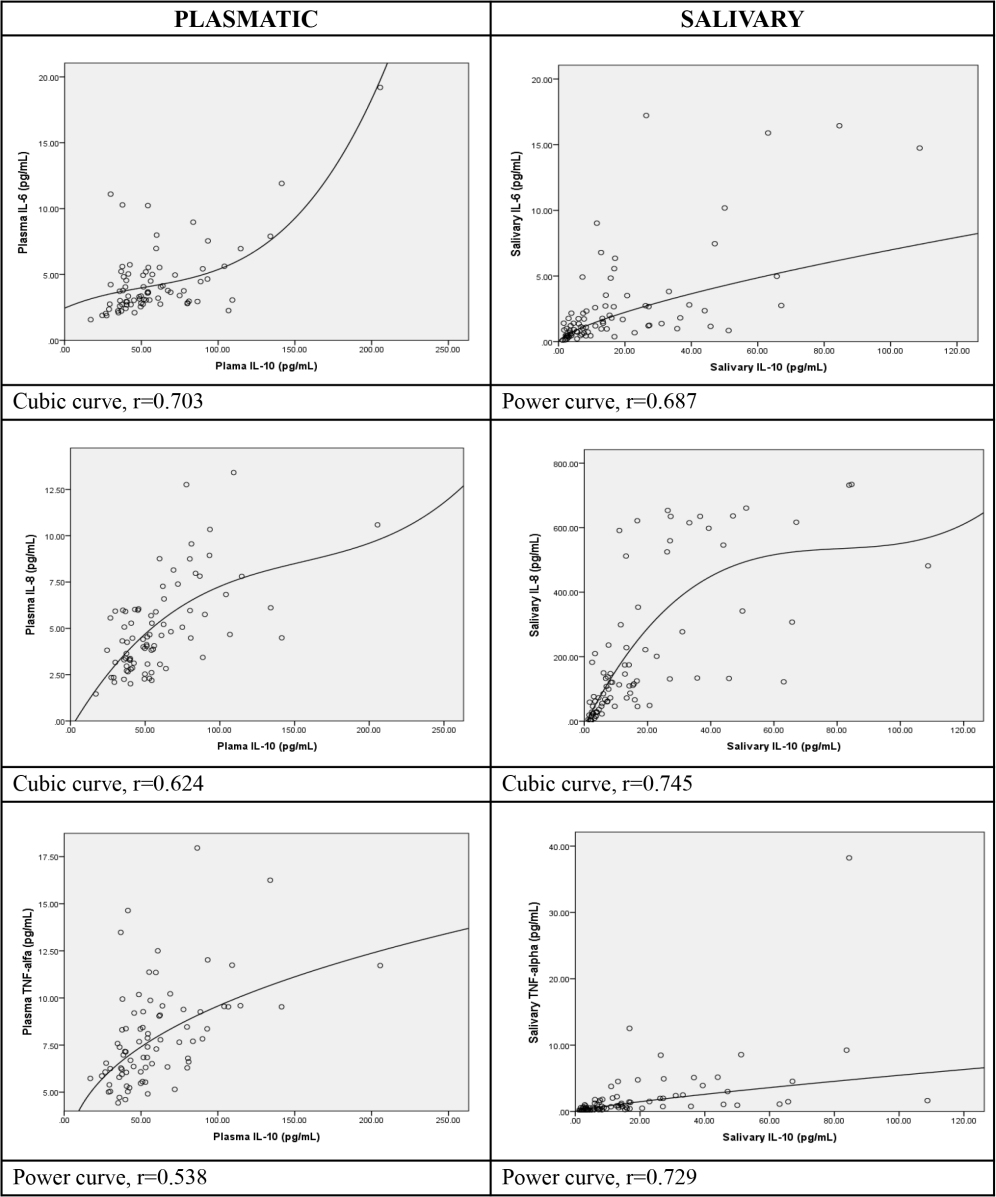
Selected Associations between Variables with the Superimposition of the Goodness-of-Fit Curve Form. Legend: Six examples of binary associations between inflammatory biomarker variables with application of the Goodness-of-Fit regressions are selected from among the 22 statistically-significant pairings in Table 3. They are chosen to illustrate the range of variation in curve form and strengths of correlation (r).

In **Fig 3**, we selected 6 examples of the goodness-of-fit curves in a parallel representation: 3 from plasma and 3 from saliva. In each panel, IL-10 is on the x-axis distribution and the y-axis distributions are for IL-6 (top panels), IL-8 (middle panels) and TNF-α (bottom panels). Those involving IL-1B have been excluded for reasons of space. Compared across biological fluids of origin, IL-10 with IL-8 shows a cubic form in the goodness-of-fit correlation; the association was stronger in the salivary pair. Similarly, a common curve-form, power, was shared by IL-10 with TNF-α across fluids, again showing the stronger association in saliva. Only in the IL-10 association with IL-6 did the curve-forms differ between anatomical compartments: for plasma, the form was cubic and for saliva, it was the power form. In this instance, plasma showed the stronger association.

### Associations by Backwards-Elimination Multiple-Regression modeling

To evaluate the independence of the prediction in the hemi-matrix of binary inter-variable Spearman correlations (**Table 4**) by backwards-elimination multiple regressions, we anchored on the cytokine IL-10, as it was the only anti-inflammatory (Th2) cytokine assayed and would theoretically be a counter-weight to the other 4 (pro-inflammatory) cytokines in the series. Restricted to the domain of only the plasma cytokines, the regression modeling produced the parsimonious model with an r^2^ value of 0.514, with IL-6, IL-8 and TNF-α remaining as predictor variables, after 2 models. When regression modeling was extended to include white blood cell count, which also had a significant association, the r^2^ value remained unchanged; it strengthened further to an r^2^ of 0.595, however, with all 11 complementary variables included in the modeling. The variables contributing determination within the model after serial modeling were plasma TNF-α, IL-6, IL-8; and salivary IL-1B. On the salivary 1L-10 side of the ledger, modeling with the remaining 4 salivary cytokines produced an r^2^ of 0.762 in the final model, in which IL-1B, IL-8 and TNF-α remained as predictors after 2 models. Adding in further values from other, non-salivary biomarkers actually weakened the magnitude of the r^2^ values of the resultant models.

**Table 4:** Coefficients of the multiple regression model for the dependent variables plasmatic and salivary IL-10 as independent variables pro-inflammatory cytokines, measured in the same body element (IL1B, IL-6, IL-8 and TNF-α).

		Unstandardized Coefficients		Standardized Coefficients			95.0% Confidence Interval for B	
No.	Model	B	Std. Error	Beta	t	Sig.	Lower Bound	Upper Bound
***Dependent variable*: *Plasmatic IL-10*** *								
2	(Constant)	-1.162	7.952		-0.146	0.884	-16.999	14.675
	Plasmatic IL-6	1.724	0.464	0.300	3.716	0.001	0.800	2.648
	Plasmatic IL-8	5.424	1.090	0.443	4.975	0.001	3.253	7.596
	Plasmatic TNF-α	3.098	1.011	0.273	3.063	0.003	1.084	5.113
***Dependent Variable*: *Salivary IL-10*** **								
2	(Constant)	4.318	1.351		3.195	0.002	1.626	7.009
	Salivary IL-1B	0.223	0.041	0.644	5.390	0.000	0.141	0.306
	Salivary IL-8	0.057	0.006	0.719	9.609	0.001	0.045	0.068
	Salivary TNF-α	-1.280	0.511	-.344	-2.504	0.014	-2.298	-0.262

*r^2^ = 0.514; r = 0.717 (n = 80)

**r^2^ = 0.762; r = 0.873 (n = 79)

## Discussion

Poor-quality water [19] and rudimentary sanitation combine with indoor oven smoke [20], diverse parasites [21] to create conditions for abundant microbes and antigens to stimulate the immune systems of low-income residents of Guatemala. Our previous studies have shown evidence of immuno-stimulation. In urban children in Guatemala City, elevated C-reactive protein (CRP), α-1-acid glycoprotein (AGP) or both were elevated in 18% of blood samples collected for hematology [22]. Although these acute-phase protein biomarkers were omitted from this study, more sophisticated and modern diagnostic indicators (cytokines) took their place. The findings on growth (Table 1) showed variation by site, from extreme stunting to mild stunting across the centers. Underweight was milder, but followed the pattern of decreased stature. Wasting was virtually non-existent.

### Comparative analysis of biomarker distributions

Leukocytosis is an elevation of the WBC above 10,500/mm^3^. It is generally associated with infections, but also arises within the metabolic syndrome, coronary heart disease, and type 2 diabetes, [23]. On their day of collection, 9 subjects (11%) had an elevated WBC.

Calprotectin is a leukocyte-derived protein found in the cytosol of inflammatory cells that has been established as a sensitive marker of intestinal inflammation [24,25]. We found 53 (61%) children with a calprotectin value >50mg/kg; this is almost twice the median value for a group of Ugandan children of a roughly comparable age and living in a correspondingly low-income setting [26].

As mentioned, cytokines are low-molecular-weight proteins that regulate immune responses, acting as mediators and messengers and are secreted by one cell to alter its own–another cell’s–function [1]. Cytokines were analyzed in two compartments: plasma and saliva. For both anatomical compartments, our reference was the reported normative-range in postmenopausal women from New York from Browne et al. [18]. With regard to the plasmatic compartment, 19 (23%) of the subjects had levels of cytokines above the reference range for IL-1B; this was 96% for IL-6; 0% for IL-8; 100% for IL-10 and 100% for TNF-α. Also, data from 223 normal-weight subjects from across Spain [27,28], which were analyzed in the same laboratory with the same method and instruments can be used as reference values. For IL-6, the mean value for Spanish children of 4.4 pg/mL compares to our median value of 3.7 pg/mL; for IL-8, their result of 1.6 pg/ml mean of Spanish children is one-third that of the Guatemalan median of 4.5 pg/mL; and for TNF-α, the Spanish reference mean of 3.1 pg/mL is less than one half of our median of 7.5 pg/mL.

Browne et al. [18] also provide normative values for the same cytokines in the salivary compartment in their study among postmenopausal women. In this anatomical compartment, 3 (4%) of our subjects have levels of cytokines above the reference range for IL-1B; this was 6% for IL-6; 14% for IL-8; 75% for IL-10; and 75% for TNF-α. In general, with some exceptions, we could find modest to high rates of elevation when compared to reference values across the biomarkers from the 4 anatomical compartments.

### Mutual interactions of cytokines within and across anatomical compartments

Of all of the biomarkers, IL-10 (plasmatic and salivary) and IL-8 (salivary) had the widest range of cross-associations, i.e. with 5 of the 11 companion biomarkers. If we isolate the interactions by type of biomarker, we see a predominance of what could be called “auto-correlation”, that is association within the same compartment, specifically within salivary or plasmatic fluids. Of the possible ten cross-associations within the cytokines of the plasmatic compartment, 7 were statistically significant, whereas all ten possible correlations were significant in the salivary compartment. Of even more interest in a biological sense might be the significant associations between biomarkers of *different* anatomical compartments, e.g. plasma versus saliva, WBC versus feces, etc. The only significant correlations between anatomical compartments were those with plasma IL-1B with salivary IL-8 and with salivary IL-10. Goodness-of-fit models were run in order to improve the association with the best curve-form; strength of association improved in less than half of the significant correlations.

We also decided to run the backwards-elimination multiple-regression model with IL-10, the only anti-inflammatory (Th2) cytokine measured in each anatomical compartment. In each model, three variables emerged to be independent predictors, with IL-8 and TNF-α commonly present as predictors in models for both anatomical compartments.

### Within-individual correspondence of cytokines in plasma and saliva

Because of the ease and innocuousness of saliva collection, especially for children, it has been recommended to exhaustively pursue its potential utility in field diagnosis [29]. In a survey of the salivary-cytokine literature, we find that the majority involve local conditions arising close to the salivary glands, such as oral inflammation [30,31] or head neck or oral, cancer [32,33]. Our observed dissociation of the cytokine response between the circulating pattern and that in the saliva, moreover, is neither unprecedented nor implausible. In the present study, we found no significant plasmatic:salivary associations for any of the 5 cytokines measured in both anatomical compartments. Browne et al. [18], in postmenopausal women, measured and associated the same 5 cytokines in both anatomical compartments with the same lack of significant association; their only finding of a significant plasmatic:salivary correlation was for IL-5. With IL-6 as the only biomarker measured, Grisius et al. [34] and Minetto et al. [35] found no significant inter-compartmental correlation in healthy North American and Italian adults, respectively; this was also the experience of Fernandez-Botrán et al. [36], investigating emotionally-abused women, again with IL-6 as their only cytokine biomarker of interest. Among 8 cytokines, including the same 5 assayed in the present study, measured concurrently in plasma and saliva in healthy female adolescents, Riis et al. [37] found only IL-1B to reach a statistically-significant correlation between compartments. In 2013, Byrne et al. [38] evaluated 11 cytokines, including 4 of the 5 cytokines measured here, across the anatomical compartments in healthy Australian adolescents; they found significant associations only for IL-2, IL-12 and INF-γ, but for none of the cytokines measured in the present study. A final–and atypical–finding comes from patients with acute myocardial infarction in an Iranian hospital; among 4 cytokines measured in both the circulation and saliva, high inter-compartmental associations (all with p values of <0.001) were found for three: IL-2; IL-6; and TNF-α [39].

Despite the lack of individual correspondence of the cytokine concentrations, we applied them on a sub-group analysis. As a post-hoc exercise to further pursue the association of the systemic (plasma) and salivary compartments to reflect the aggregation of inflammation, we created a cumulative rank-order score for the 5 cytokines in each biological fluid. Separately, for each anatomical compartment, the sum of the rank (in ascending order) of each child in each of the 5 distributions: that is, the child with the lowest cytokine in each anatomical compartment was ranked as 1, and the individual with the highest value was ranked number 80, the number of children that had data for all cytokines. Thus, the combined composite scale could run from a high of 400 (the same individual was most inflamed for all 5 cytokines) to a low of 5 (the same individual was least inflamed for all 5 cytokines). As calculated, the individual cumulative rank-order score values ranged from 35 to 361 for the plasma biomarkers and 15 to 398 for the salivary. Even with this composite scoring of all cytokines, the Spearman correlation coefficient for the plasma cumulative rank-order score with its salivary counterpart still showed an insignificant association (r = -0.036, p = 0.749).

Because circulating cytokines arise from adipose, hepatic and peripheral and tissue-fixed white blood cells [1], they would respond to systemic immuno-stimulation; by contrast, the salivary cytokines arise in salivary and oral sites governed by local events in and around the buccal cavity [40]. It would seem that systemic immune response has low penetrance to the sites of salivary cytokines’ origin.

### Hierarchical, group-wise relationships of cytokine biomarkers

Although there is scant inter-compartmental correspondence at the individual level, that fact does not dismiss all of the possibilities. The question still remains as to whether salivary cytokines can be used as a proxy for those in the circulation on a *group-wise* basis. A survey in Sweden approached the utility of salivary cytokines as an epidemiological tool for screening for systemic diseases [41]. They considered tobacco smoking and 8 selected systemic disease conditions as self-reported by 1000 adults in southern Sweden. The cytokine biomarkers were IL-1B, IL-6, IL-8 and TNF-α. For histories of heart surgery, heart disease, hypertension, diabetes and mental disorders, there were no associations with elevated salivary cytokines. Moreover, with salivary IL-6 and TNF-α, there were no association with any of the selected conditions. However, salivary IL-8 was elevated significantly in those reporting being smokers and having a history of tumors, bowel diseases and muscle and joint disorders. The latter was also associated independently with elevation of IL-1B. They suggest a certain utility of salivary cytokine biomarkers in population epidemiology. Plasma cytokines were not concurrently measured. Similar explorations of salivary cytokines with systemic diseases have been reported individually including: inflammatory bowel diseases [42]; pediatric type 1 diabetes [43]; obesity-related sleep apnea [44]; and cutaneous lichen planus [45]. Cytokines elevations were found for IL-8 among 90 salivary proteins after total body irradiation [46], similarly anti-inflammatory cytokine, IL-10, rose in saliva of airway-disease patients when exposed to thermal sulfur water bath therapy [47]. Finally, monitoring of diverse salivary biomarkers during 520 days of simulated space travel to Mars, showed no change in the 4 cytokines evaluated: IL-2, IL-6, TNF-α and INF-γ [48].

Research surrounding stress responses and the anti-inflammatory cytokine, IL-6 has been prominent. Reviewing the literature, Slavish and Graham-Engeland [49] conclude that the findings are currently inconsistent. Salivary IL-6 has been elevated in response to a social stress test [50], viewing disgusting visual images [51], spinning gyrations on a rotary device [35], and a psychiatric counseling visit [52]; this response was not seen, however, exercising to the point of exhaustion [35]. The elevation was also associated with active post-traumatic stress syndrome (PTSD) associated with spousal abuse in women [52]. In three situations in two studies, circulating IL-6 was simultaneously evaluated. The salivary and plasmatic responses corresponded with PTSD [52] and rotator stress [35], but not with exhausting exercise [35].

To examine this group-wise phenomenon in our own study setting, we calculated medians of the previously-described composite cytokine rank-order scores. We analyzed them by daycare site and by each body fluid (**Table 5**). Interestingly, in the sense of a common trend, the *relative* hierarchy was common across anatomical compartments, with the highest cytokine rankings clustered in the rural zone for both composites scores; the intermediate values are in the marginal-urban area; and the lowest rankings are seen in the semi-urban setting. The Kruskal-Wallis test, however, failed to find a significant difference among the sites.

**Table 5:** Median Cytokine Cumulative Rank-Score by setting.

		Plasmatic Cytokines	Salivary Cytokines
Setting	N	median score (95% CI)	
Semi-Urban (A)	20	172 (151–231)	134 (105–217)
Marginal-Urban (B)	38	186 (152–225)	184 (162–233)
Rural-(C)	22	227 (191–243)	246 (194–260)
p-value		0.349*	0.090*

*Comparison among settings using Kruskal-Wallis test.

### Strengths and limitations of the study

We acknowledge certain strengths and weaknesses of design and execution of the study. The principal strength is that it deals with childhood, with children of preschool age, in a context of poor hygienic environment. Also related to young age are two collection methods, fecal and salivary, which are non-invasive and do not require the extraction of blood. In theory, the settings with similar dietary offering should control one important variable, and narrow overall variance. An additional strength is the possibility to relate a large variety of inflammatory biomarkers from different anatomical compartments. A very important methodological strength is that we can compare our cytokine values with those generated with the identical multiplexing method [18], and, in the case of the Spanish children [27,28], a similar age-range and the same laboratory and equipment in Granada.

The foremost limitation of the study is that our sample-size of 87 is modest, and the analyses by separate daycare site may have lacked statistical power to fully capture differences by geographical setting **(Table 5)**. In addition, we were not able to measure the two most commonly used biomarkers of inflammation, CRP and APG, if not simply to relate them to the more sophisticated cytokine panels. Moreover, saliva is a viscous matrix. At least for an ELISA method, Dafar et al., [53] found that extraction with sodium dodecyl sulfate (SDS) improved the detection of one of the cytokines of interest, salivary IL-8; whether this applies to the Milliplex method as well is not known. Thomas et al. [54] examined day-to-day fluctuation in salivary IL-1B, IL-6 and TNF-α finding coefficients of variation of up to 200%. As our design called for only a single day’s collection, we can perhaps understand how the internal correlation among salivary cytokines could be so well coordinated while their associations with biomarkers in other anatomical compartments could be so poor. One could generalize this point to project that associations with factors external to the subjects might only be revealed with multiple repetitions of the measurements in the same individuals.

## Conclusion

Twelve biomarkers of inflammation from the whole blood, fecal, plasma and salivary compartments showed a diverse array of findings and interactions among low-income preschool children from the western highlands of Guatemala. In general, the indicators were elevated above reference levels, suggesting a response to the microbial and antigenic milieu of the poorly hygienic surroundings in which they live. The marker of fecal inflammation exhibited no interaction with any biomarker in any other domain. White cells showed a modest interaction, associating positively with three circulating cytokines. Similarly modest were the cross-associations for cytokines between plasma and saliva, with plasmatic IL-1B having two significant −and negative− associations. Within-compartment, however, the plasma and salivary cytokines showed a vigorous mutual interaction. The prominence of the anti-inflammatory cytokine IL-10 in both plasma and saliva is consistent with its counter-regulatory role in the face of elevated pro-inflammatory cytokines.

## Supporting Information

S1 Checklist STROBE Statement of items included in this cross-sectional study. (DOC)

## References

[1] Noakes PS, Michaelis LJ (2013) Innate and Adaptive Immunity In: Calder P, Yaqoob P, editors. Diet, Immunity and Inflammation. Cambridge: Woodhead Publishing Limited pp. 3–33. 10.1007/978-0-387-39241-7_2

[2] Keusch G, Farthing MJ (1986) Nutrition and infection. Annu Rev Nutr 6: 131–154. 3524614 10.1146/annurev.nu.06.070186.001023

[3] FAO (2013) Panorama de la Seguridad Alimentaria y Nutricional en América Latina y el Caribe.

[4] Berngard SC, Berngard JB, Krebs NF, Garcés A, Miller LV, Westcott J, et al (2013) Newborn length predicts early infant linear growth retardation and disproportionately high weight gain in a low-income population. Early Hum Dev 89: 967–972. 10.1016/j.earlhumdev.2013.09.008 24083893 PMC3859373

[5] Solomons NW, Vossenaar M, Chomat A-M, Doak CM, Koski KG, Scott M, et al. (2014) Stunting at birth: recognition of early-life linear growth failure in 2 the western highlands of Guatemala. Public Health Nutr, available on CJO2014. 10.1017/S136898001400264X PMC10271386 26017476

[6] Victora CG, de Onis M, Hallal PC, Blössner M, Shrimpton R (2010) Worldwide timing of growth faltering: revisiting implications for interventions. Pediatrics 125: e473–e480. 10.1542/peds.2009-1519 20156903

[7] Dewey KG, Begum K (2011) Long-term consequences of stunting in early life. Matern Child Nutr 7 Suppl 3: 5–18. 10.1111/j.1740-8709.2011.00349.x 21929633 PMC6860846

[8] Rytter MJH, Kolte L, Briend A, Friis H, Christensen VB (2014) The immune system in children with malnutrition-a systematic review. PLoS One 9: e105017 10.1371/journal.pone.0105017 25153531 PMC4143239

[9] Beisel WR (1995) Herman Award malnutrition-from. Am J Clin Nutr 62: 813–819. 7572715 10.1093/ajcn/62.4.813

[10] Coates ME, Fuler R, Harrison GF, Lev M, Sufolk SF (1963) A comparison of the growth of chicks in the Gustafson germ-free aparatus and in conventional environment, with and without dietary suplements of penicilin. Br J Nutr 17: 141–150. 14021819 10.1079/bjn19630015

[11] Hill DC, Branion HD, Slinger SI, Anderson GW (1952) Influence of environment on the growth response of chicks to penicilin. Poult Sci 32: 464–466.

[12] Roura E, Homedes J, Klasing KC (1992) Nutrient Metabolism Prevention of Immunologie Stress Contributes to the Growth-Permitting Ability of Dietary Antibiotics in Chicks. J Nutr: 2383–2390. 10.1093/jn/122.12.2383 1453223

[13] Solomons NW, Mazariegos M, Brown KH, Klasing K (1993) The underprivileged, developing country child: environmental contamination and growth failure revisited. Nutr Rev 51: 327–332. 8108032 10.1111/j.1753-4887.1993.tb03758.x

[14] Pfister K, Ramel S (2014) Linear growth and neurodevelopmental outcomes. Clin Perinatol 41: 309–321. 10.1016/j.clp.2014.02.004 24873834

[15] Ngure FM, Reid BM, Humphrey JH, Mbuya MN, Pelto G, Stoltzfus RJ, et al (2014) Water, sanitation, and hygiene (WASH), environmental enteropathy, nutrition, and early child development: making the links. Ann N Y Acad Sci 1308: 118–128. 10.1111/nyas.12330 24571214

[16] Prendergast AJ, Rukobo S, Chasekwa B, Mutasa K, Ntozini R, Mbuya MNN, et al (2014) Stunting is characterized by chronic inflammation in Zimbabwean infants. PLoS One 9: e86928 10.1371/journal.pone.0086928 24558364 PMC3928146

[17] Odiere MR, Scott ME, Leroux L, Dzierszinski FS, Koski KG (2013) Maternal Protein Deficiency during a Gastrointestinal Nematode Infection Alters Developmental Profile of Lymphocyte Populations and Selected Cytokines in Neonatal Mice. J Nutr: 143: 100–107. 10.3945/jn.112.160457 23190758

[18] Browne RW, Kantarci A, LaMonte MJ, Andrews C a, Hovey KM, Falkner KL, et al (2013) Performance of multiplex cytokine assays in serum and saliva among community-dwelling postmenopausal women. PLoS One 8: e59498 10.1371/journal.pone.0059498 23577067 PMC3618114

[19] Sobel J, Mahon B, Mendoza CE, Passaro D, Cano F, Baier K, et al (1998) Reduction of Fecal Contamination of Street-Vended Beverages in Guatemala by a Simple System for Water Purification and Storage, Handwashing, and Beverage Storage. Am J Trop Med Hyg. 59: 380–387. 9749629 10.4269/ajtmh.1998.59.380

[20] Boy E, Bruce N, Delgado H (2002) Birth weight and exposure to kitchen wood smoke during pregnancy in rural Guatemala. Environ Health Perspect 110: 109–114. 11781172 10.1289/ehp.02110109 PMC1240700

[21] Duffy T (2011) Giardiasis in children attending daycare centers in Guatemala and the therapeutic potential of ganglioside. Thesis for Master of Science in Nutrition and Metabolism. Agricultural, Food & Nutritional Science, University of Alberta, Edmonton, Canada; 2011.

[22] Beard JL, Murray-kolb LE, Rosales FJ, Solomons NW, Angelilli ML (2006) Interpretation of serum ferritin concentrations as indicators of total-body iron stores in survey populations: the role of biomarkers for the acute phase response. Am J Clin Nutr. 84: 1498–1505. 17158435 10.1093/ajcn/84.6.1498

[23] Chen W, Srinivasan SR, Xu J, Berenson GS (2010) Black-white divergence in the relation of white blood cell count to metabolic syndrome in preadolescents, adolescents, and young adults: the Bogalusa Heart Study. Diabetes Care 33: 2474–2476. 10.2337/dc10-0619 20798336 PMC2963517

[24] Van Rheenen PF, Van de Vijver E, Fidler V (2010) Faecal calprotectin for screening of patients with suspected inflammatory bowel disease: diagnostic meta-analysis. Bmj 341: c3369–c3369. 10.1136/bmj.c3369 20634346 PMC2904879

[25] Burri E, Beglinger C (2014) The use of fecal calprotectin as a biomarker in gastrointestinal disease. Expert Rev Gatroenterology Hepatol 8: 197–210. 10.1586/17474124.2014.869476 24345070

[26] Hestvik E, Tumwine JK, Tylleskar T, Grahnquist L, Ndeezi G, Kadu-Mulindwa DH, et al (2011) Faecal calprotectin concentrations in apparently healthy children aged 0–12 years in urban Kampala, Uganda: a community-based survey. BMC Pediatr 11: 9 10.1186/1471-2431-11-9 21284894 PMC3039585

[27] Olza J, Aguilera CM, Gil-Campos M, Leis R, Bueno G, Martínez-Jiménez MD, et al (2012) Myeloperoxidase is an early biomarker of inflammation and cardiovascular risk in prepubertal obese children. Diabetes Care 35: 2373–2376. 10.2337/dc12-0614 22912422 PMC3476926

[28] Olza J, Aguilera CM, Gil-Campos M, Leis R, Bueno G, Valle M, et al (2014) Waist-to-height ratio, inflammation and CVD risk in obese children. Public Health Nutr 1: 1–8. 10.1017/S1368980013003285 PMC10282624 24476930

[29] Smith D, Shalmiyeva I, Deblois J, Winke M (2012) Use of salivary osmolality to assess dehydration. Prehospital Emerg Care 16: 128–135. 10.3109/10903127.2011.614044 21950414

[30] Cvikl B, Lussi A, Moritz A, Sculean A, Gruber R (2014) Sterile-filtered saliva is a strong inducer of IL-6 and IL-8 in oral fibroblasts. Clin Oral Invest 19: 385–399. 10.1007/s00784-014-1232-3 25115993

[31] Abdel-Haq A, Kusnierz-Cabala B Darczuk D, Sobuta E, Dumnicka P, Wojas-Pelc A, et al (2014) Interleukin-6 and neopterin levels in the serum and saliva of patients with Lichen planus and oral Lichen planus. J oral Pathol Med 43: 734–739. 10.1111/jop.12199 24935446

[32] Hamzavi M, Tadbir AA, Rezvani G, Ashraf MJ, Fattahi MJ, Khademi B, et al (2013) Tissue Expression, Serum and Salivary Levels of IL-10 in Patients with Head and Neck Squamous Cell Carcinoma. Asian Pacific J Cancer Prev 14: 1681–1685. 10.7314/APJCP.2013.14.3.1681 23679256

[33] Prasad G, McCullough M (2013) Chemokines and cytokines as salivary biomarkers for the early diagnosis of oral cancer. Int J Dent 2013: 813756 10.1155/2013/813756 24376459 PMC3860143

[34] Grisius M, Bermudez D, Fox P (1997) Salivary and serum interleukin 6 in primary Sjögren’s syndrome. J Rheumatol 24: 1089–1091. 9195514

[35] Minetto M, Gazzoni M, Lanfranco F, Baldi M, Saba L, Pedrola R, et al (2007) Influence of the sample collection method on salivary interleukin-6 levels in resting and post-exercise conditions. Eur J Appl Physiol 101: 249–256. 17569075 10.1007/s00421-007-0484-x

[36] Fernandez-Botran R, Miller J, Burns V, Newton T (2011) Correlations Among Inlamatory Markers in Plasma, Saliva, and Oral Mucosal Transudate in Postmenopausal Women with Past Intimate Partner Violence. Brain Behav Inmmun 25: 314–321. 10.1016/j.bbi.2010.09.023 20888902 PMC3025073

[37] Riis J, Out D, Dorn L, Beal S, Denson L, Pabst S, et al (2014) Salivary cytokines in healthy adolescent girls: Intercorrelations, stability, and associations with serum cytokines, age, and pubertal stage. Develomental Psychobiol 56: 797–811. 10.1002/dev.21149 23868603 PMC8136359

[38] Byrne M, O’Brien-Simpson N, Reynolds E, Walsh K, Laughton K, Waloszek JM, et al (2013) Acute phase protein and cytokine levels in serum and saliva: a comparison of detectable levels and correlations in a depressed and healthy adolescent sample. Brain Behav Inmmun 34: 154–175. 10.1016/j.bbi.2013.08.010 23999491

[39] Assareh A, Haybar H, Yoosefi H, Bozorgmanesh M (2013) Bedside-Friendly Prediction for Presence of Post-Myocardial lnfarction Systolic Dysfunction Using Multimarker Panel: Integrating Salivary Diagnostics into Clinical Practice. Korean Circ J 43: 246–254. 10.4070/kcj.2013.43.4.246 23682284 PMC3654112

[40] Brogden K, Johnson G, Vincent S, Abbasi T, Vali S (2013) Oral inflammation, a role for antimicrobial peptide modulation of cytokine and chemokine responses. Expert Rev Anti-inefective Ther 11: 1097–1113. 10.1586/14787210.2013.836059 24124799

[41] Rathnayake N, Akerman S, Klinge B, Lundegren N, Jansson H, Tryselius Y, et al (2013) Salivary biomarkers for detection of systemic diseases. PLoS One 8: e61356 10.1371/journal.pone.0061356 23637817 PMC3634781

[42] Said HS, Suda WA, Nakagome SH, Chinen HI, Oshima KE, Kim S, et al (2014) Dysbiosis of Salivary Microbiota in Inflammatory Bowel Disease and Its Association With Oral Immunological Biomarkers. DNA Res 21: 15–25. 10.1093/dnares/dst037 24013298 PMC3925391

[43] Dakovic D, Colic M, Cakic S, Mileusnic I, Hajdukovic Z, Stamatovic N (2013) Salivary interleukin-8 levels in children suffering from type 1 diabetes mellitus. J Clin Pediatr Dent 37: 377–380. 24046985 10.17796/jcpd.37.4.l135531h4542gj66

[44] Nizam N, Basoglu O, Tasbakan M, Nalbantsoy A, Buduneli N (2014) Salivary cytokines and the association between obstructive sleep apnea syndrome and periodontal disease. J Periodontol 85: e251–e258. 10.1902/jop.2014.130579 24410293

[45] Abdel-Haq A, Kusnierz-Cabala B, Darczuk D, Sobuta E, Dumnicka P, Wojas-Pelc A, et al (2014) Interleukin-6 and neopterin levels in the serum and saliva of patients with Lichen planus and oral Lichen planus. J Oral Pathol Med 43: 734–739. 10.1111/jop.12199 24935446

[46] Moore H, Ivey R, Voytovich U, Lin C, Stirewalt D, Pogosova-Agadjanyan EL, et al (2014) The human salivary proteome is radiation responsive. Radiat Reseach 181: 521–530. 10.1667/RR13586.1 24720749 PMC4110973

[47] Prandelli C, Parola C, Buizza L, Delbarba A, Marziano M, Salvi V, et al (2013) Sulphurous thermal water increases the release of the anti-inflammatory cytokine IL-10 and modulates antioxidant enzyme activity. Int J Immunopathol Pharmacol 26: 633–646. 24067460 10.1177/039463201302600307

[48] Yi B, Rykova M, Feuerecker M, Jäger B, Ladinig C, Basner M, et al (2014) 520-d Isolation and confinement simulating a flight to Mars reveals heightened immune responses and alterations of leukocyte phenotype. Brain Behav Immun 40: 203–210. 10.1016/j.bbi.2014.03.018 24704568

[49] Slavish DC, Graham-Engeland JE, Smyth JM, Engeland CG (2015) Salivary markers of inflammation in response to acute stress. Brain Behav Immun 44: 253–269. 10.1016/j.bbi.2014.08.008 25205395 PMC4275319

[50] Izawa S, Sugaya N, Kimura K, Ogawa N, Yamada K, Shirotsuki K, et al (2013) An increase in salivary interleukin-6 level following acute psychosocial stress and its biological correlates in healthy young adults. Biol Psychol 94: 249–254. 10.1016/j.biopsycho.2013.06.006 23831278

[51] Ersche K, Hagan C, Smith D, Abbott S, Jones P, Apergis-Schoute AM, et al (2014) Aberrant Disgust Responses and Immune Reactivity in Cocaine-Dependent Men. Biol Psychiatry 75: 140–147. 10.1016/j.biopsych.2013.08.004 24090796 PMC3898808

[52] Newton T, Fernandez-Botran R, Miller J, Burns V (2014) Interleukin-6 and soluble interleukin-6 receptor levels in posttraumatic stress disorder: associations with lifetime diagnostic status and psychological context. Biol Psychol 99: 150–159. 10.1016/j.biopsycho.2014.03.009 24695006 PMC4059765

[53] Dafar A, Rico P, Işık A, Jontell M, Cevik-Aras H (2014) Quantitative detection of epidermal growth factor and interleukin-8 in whole saliva of healthy individuals. J Immunol Methods 408: 46–51. 10.1016/j.jim.2014.04.013 24816468

[54] Thomas M, Branscum A, Miller C, Ebersole J, Al-Sabbagh M, Schuster JL, (2014) Within-subject variability in repeated measures of salivary analytes in healthy adults. J Periodontol 80: 1146–1153. 10.1902/jop.2009.080654 PMC4131719 19563296

